# Impact of altering the available food options on selection: Potential mediation by social norms

**DOI:** 10.1016/j.appet.2021.105245

**Published:** 2021-09-01

**Authors:** Rachel Pechey, Natasha Clarke, Emily Pechey, Minna Ventsel, Gareth J. Hollands, Theresa M. Marteau

**Affiliations:** a Behaviour and Health Research Unit, Institute of Public Health, University of Cambridge, Cambridge, UK; b Nuffield Department of Primary Care Health Sciences, University of Oxford, OX2 6GG, UK

**Keywords:** Food, Availability, Mechanism, Social norms

## Abstract

Increasing the availability of lower-energy foods increases their selection. The current studies examine the extent to which this effect could be mediated by social norms – assessed by perceived popularity of foods – which may be implied by their relative availability. Study 1 (Online): 2340 UK adults estimated the perceived popularity of products. Participants were randomised to see photos of cafeteria shelves varying in the availability of lower-energy options (^1^/_4_ lower-energy; ^1^/_2_ lower-energy; ^3^/_4_ lower-energy) and fullness of shelves (fuller; emptier). Study 2 (Laboratory): 139 English adults were asked to select a snack. Participants were randomised to select from trays varying in the availability of the lower-energy option (^1^/_3_ lower-energy; ^2^/_3_ lower-energy) and fullness of tray (fuller; emptier). In Study 1, evidence for an interaction was found, such that when shelves were fuller, a higher proportion of lower-energy options led to greater perceived popularity of lower-energy products (^1^/_4_ lower-energy: 40.9% (95%CIs: 40.1,41.8); ^3^/_4_ lower-energy: 47.2% (46.3,48.0)), whereas when shelves were emptier, a higher proportion of lower-energy options led to lower perceived popularity (^1^/_4_ lower-energy: 48.4% (47.5,49.2); ^3^/_4_ lower-energy: 39.2% (38.3,40.0)). In Study 2, when the tray was fuller, participants were more likely – albeit non-significantly – to select a lower-energy snack when ^2^/_3_ of the available snacks were lower-energy (35.7% (18.5,52.9)) than when ^1^/_3_ were lower-energy (15.4% (4.2,26.5)). For emptier trays, lower-energy selections decreased as the relative availability of lower-energy snacks increased (^1^/_3_ lower-energy snacks: 36.0% (17.9,54.1); ^2^/_3_ lower-energy snacks: 27.8% (13.9,41.7)). These studies provide novel evidence that social norms may mediate the impact of availability on food selection. In addition, they suggest that the effect of availability may be moderated by display layout through its impact on perceived product popularity.

## 1 Background

Increasing the availability of lower energy and more plant-based foods increases their selection (Garnett et al., 2019; Hollands et al., 2019; Pechey et al., 2019a; Pechey & Marteau, 2018). However, very little work has been conducted to try to explore the potential mechanisms underlying this effect (Pechey et al., 2020; Pechey et al., preprint). Such possible mechanisms include prior preferences (with selections reflecting individuals’ most-preferred option from the available range), increased availability being perceived to reflect greater selection of highly stocked options by others (leading to updating of social norms), and/or attention being more likely to be drawn to the increased options (Pechey et al., 2020). The focus of this work is the potential role of social norms – examined through perceptions of popularity – in explaining effects of availability interventions on behaviour.

**Fig. 1:**
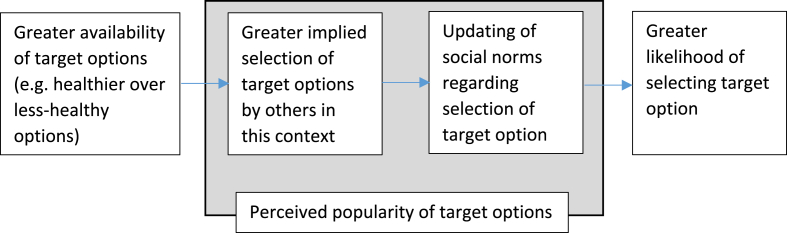
Logic model of role of perceived popularity in pathway from availability to selection, based on pathway 2 in Pechey et al. (Pechey et al., 2020).

The perceived popularity of particular options might impact on behaviour via updating of social norms relating to selection or consumption of these products (see logic model (Fig. 1), based on pathway 2 in Pechey et al., 2020). When presented with information about the behaviour of others (i.e. information relating to social norms), individuals are more likely to behave in a similar way (e.g. (Mortensen et al., 2017, Sparkman and Walton, 2017)). Deutsch and Gerard (Deutsch & Gerard, 1955) proposed individuals follow social norms in order to: (a) enhance affiliation with social group – i.e. they want to be liked – and (b) to perform the ‘correct’ behaviour. Indeed, people may be more likely to follow norms if they specifically refer to a group salient to the individual (Cruwys et al., 2015) and if they do not feel socially accepted (Robinson et al., 2011). Such modelling behaviour goes beyond mere imitation, involving an emotional component such as the desire to avoid social sanctions that may be imposed on those who do not follow such norms (Baumeister & Leary, 1995).

In the context of dietary behaviour, when individuals have knowledge of others' behaviour in the same setting, they are more likely to make similar selections or consume similar amounts (Pliner & Mann, 2004; Prinsen et al., 2013; Salmon et al., 2014). Descriptive social norms – encompassing people's beliefs about how common behaviours are in general or among individuals salient to them – may be key. These are correlated with healthy eating behaviours (Ball et al., 2010), whereas injunctive norms – beliefs about what other people expect or approve of with regard to these behaviours – are not (Lally et al., 2011). Providing social normative information may alter behaviour via changing expected liking for targeted foods and beverages (Robinson & Higgs, 2012), reflected in reward-related brain activity (Izuma & Adolphs, 2013; Nook & Zaki, 2015). Studies manipulating social norm information often make others' behaviour explicit – e.g. showing individuals lists that outline “previous participants' selections” – but fewer studies have looked at the impact of implied popularity or implied social norms on behaviour.

One study has shown that environmental cues in the form of empty wrappers indicating previous participants’ behaviour influence selections, with the presence of empty wrappers increasing selections of that product (Prinsen et al., 2013). It is possible that such implied popularity might also influence behaviour in purchasing contexts in a similar manner. For example, if a vending machine has few units of a particular type of product remaining, emptier slots within the machine might imply greater popularity. To our knowledge, this has not yet been tested. Moreover, it is unclear whether the opposite pattern might also be seen in cafeterias or supermarkets. For example, given awareness of the idea of supply (availability) and demand in commercial enterprises such as cafeterias or supermarkets, greater presence of certain options – e.g. a greater number of types of chocolate bars vs. types of lower energy snack bars – might imply greater popularity of these products in these contexts.

The aim of this set of studies is to provide the first exploration of the role of perceived popularity – as a marker of descriptive social norms – as a possible mechanism underlying the effects of availability interventions. The paper focuses on relative availability (Pechey et al., 2020) – i.e. altering the proportion of lower energy vs. higher energy options, while keeping the overall number of options constant – given that the relative popularity of each of these categories is hypothesised to depend to a greater extent on relative, rather than absolute, availability. In particular, these studies will address the extent to which (1)1 the relative availability of a food impacts on its perceived popularity (i.e. perceptions regarding consumption or selection of that food by others); and (2) any difference in popularity implied by the relative availability of a food alters the selection of that food.

## 2 Methods

### 2.1 Studies

Study 1 investigates whether altering the relative availability of lower energy food affects the perceived popularity of these lower energy foods (i.e. Question 1 above) in an online setting. Participants were also asked to select a food item in this study, to provide some preliminary evidence as to whether perceived popularity impacts on behaviour. To do so, however, required participants to explicitly state their perceptions regarding popularity before making their food selection, whereas when availability has been found to impact on behaviour in previous studies such perceptions would not have been highlighted.

Study 2 therefore explores the impact of manipulating the perceived popularity of lower energy (over higher energy) options on food selection (i.e. Question 2 above) – without making these perceptions regarding popularity explicit to participants – to match the contexts where effects of availability on food selections were observed in previous studies.

Both studies were pre-registered on the Open Science Framework (https://osf.io/c2amf) and ISRCTN (http://www.isrctn.com/ISRCTN10512908). Ethical approval was obtained from the University of Cambridge Psychology Research Ethics Committee (Ref: PRE.2019.100).

## 3 Study 1: Online study

### 3.1 Study 1 Aims

1. To investigate the extent to which increasing the relative availability of lower energy (over higher energy) food impacts on perceived popularity of these foods
2. To inform Study 2, by conducting an initial exploration of the extent to which altering the perceived popularity of lower energy and higher energy foods impacts on selection of lower energy foods

#### 3.1.1 Primary Research Questions (aim 1)

1. To what extent does the relative availability of lower energy vs. higher energy foods impact on perceived popularity of these products?
2. To what extent does manipulating whether the shelves displaying available items are fuller vs. emptier impact on perceived popularity of these products?

#### 3.1.2 Secondary Research Questions (aim 2)

3. To what extent does the perceived popularity of lower energy vs. higher energy foods alter the selection of lower energy foods?
4. To what extent does manipulating availability at the product-level (i.e. the number of units of a product, e.g. 4 vs. 8 cans of a brand of soft drink) rather than the category-level (i.e. the number of unique products, e.g. 2 vs. 4 brands of soft drinks) alter the impact of relative availability on selection of lower energy foods?

##### 3.1.2.1 Participants

A UK-representative sample of adults was recruited from a market research agency panel (Dynata UK), with quotas set by highest educational qualification (higher: 2 or more A Levels or higher; lower: up to GCSE-level education or equivalent). Those who were studying for A-levels were excluded from participation, to avoid classifying those who had not yet achieved these qualifications in the lower education group. Participants who failed an attention check question or who completed the survey in less than 30% of the median time (speeders) were excluded.

Sample size calculations allowed for a minimum of four trials (photos of food/drink displays), with large variability of level-1 residuals and level-1 coefficient (both set to 2), and a small-medium standardised effect size (d = 0.27 – equating to half the difference observed between the equal numbers of lower energy and higher energy options and predominantly higher energy options conditions in a previous laboratory study (Pechey et al., 2021)). The Optimal Design sample size calculator, set to alpha of 0.01 (to adjust for multiple comparisons) and with power of 0.8, suggested a sample size of 772 (i.e. 386 per group). Accounting for the six groups (3 × 2 availability by shelf-fullness conditions) gives a total sample size of 2316, rounded to 2340 to allow for some missing data.

Of those completing the online study and correctly answering the attention check question (n = 2343; 51 participants who incorrectly answered the attention check were screened out and did not count towards quotas), 12 were excluded as speeders. This left a final sample size of 2331 participants.

##### 3.1.2.2 Design

Online study, with 3 × 2 between-subjects availability x shelf-fullness conditions, and an additional within-subjects manipulation (product-level vs. category-level changes). The three between-subjects availability conditions were: ^1^/_4_ lower energy and ^3^/_4_ higher energy; ^1^/_2_ lower energy and ^1^/_2_ higher energy; ^3^/_4_ lower energy and ^1^/_4_ higher energy.

Two between-subjects shelf-fullness conditions were included: Shelves-fuller vs. shelves-emptier. The shelves-emptier manipulations displayed the same products as in the shelves-fuller condition with ^1^/_2_ lower energy and ^1^/_2_ higher energy options, but the number of units of the products varied by condition. As such when 1/4 of items available were lower energy, more units of lower energy products were removed and the shelf space for lower energy options was emptier than for higher energy options, which could imply that a greater number of people had previously selected these options (see Table 1).

**Table 1:** Between-subjects study conditions, with example images.

		Relative availability		
		^1^/_4_ lower energy & ^3^/_4_ higher energy	^1^/_2_ lower energy & ^1^/_2_ higher energy	^3^/_4_ lower energy & ^1^/_4_ higher energy
Shelf-fullness	Shelves-fuller	Group 1	Group 2	Group 3
	Shelves-emptier	Group 4	Group 5	Group 6

**Fig. 2:**
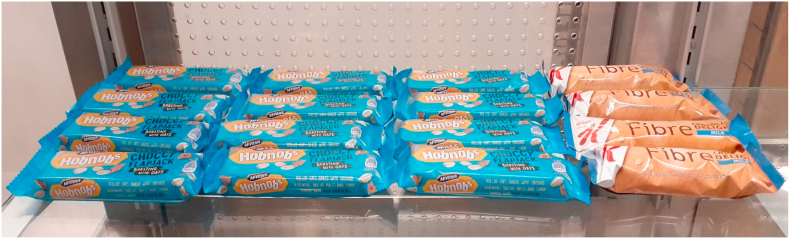
Example of a product-level manipulation.

Two manipulation-level conditions were also included (within-subjects): Category-level (changing range of products) vs. product-level (changing the number of units of two representative products). See Fig. 2 for an example of a product-level manipulation; Table 1 for an example of a category-level manipulation.

Trials presented photos showing a range of pre-packaged food and drink categories, including cold drinks, sweet snacks, and savoury snacks. Each participant viewed each set of products once, and was randomly allocated to one of the six conditions for each product set.

Photos for each trial were taken in a real-world purchasing context (a worksite cafeteria), given it is unclear whether social norms would play a role if items were displayed in the absence of any context, e.g. in previous online studies such as Pechey & Marteau (2018).

##### 3.1.2.3 Selection of products for images

For category-level changes, each participant saw four images: Cans of diet/sugar-free drinks vs. cans of sugary drinks; Cereal/snack bars vs. chocolate bars; Lentil/chickpea crisps vs. potato crisps; and Popcorn vs. crisps. For product-level changes, participants again each saw four images: Bottles of low calorie Lucozade Sport (Orange) vs. standard Lucozade Sport (Orange); Special K Fibre bar (Milk Chocolate) vs. Hobnobs flapjack (Milk chocolate); Snack-a-Jacks (Salt & Vinegar) vs. Mini Cheddars (Original); and Popchips (BBQ) vs. Hula Hoops Big Hoops (BBQ).

Each product – in both the category-level or product-level manipulations – met the study definition of a lower energy or higher energy product. Healthiness of options was defined to match that used in previous studies. For snacks (Pechey & Marteau, 2018), lower energy options had 100 kcal or less per pack, whereas higher energy options had 200 kcal or more per pack. For drinks (Pechey et al., 2019b), lower energy options had less than 2.5 g of sugar per 100 ml, whereas higher energy options had 2.5 g or more of sugar per 100 ml. All products were single-serve snacks or drinks.

Lower energy food and drink options were all on sale on the websites of the three largest UK supermarkets (Tesco, Sainsbury's and Asda) to ensure they were likely to be recognisable and familiar to participants. Drinks were directly matched by brand and flavour for lower energy (diet) vs. higher energy (full-sugar) options. For sweet and savoury snacks, the most popular brand names were excluded for higher energy options (Savoury snacks: Walkers, McCoys, Doritos (Hyslop, 2017); Sweet Snacks: e.g. Cadbury's, Galaxy, Mars, Nestle (Kitkat) (Statista, 2015) to minimise differences in familiarity between lower energy and higher energy options.

##### 3.1.2.4 Procedure

**Fig. 3:**
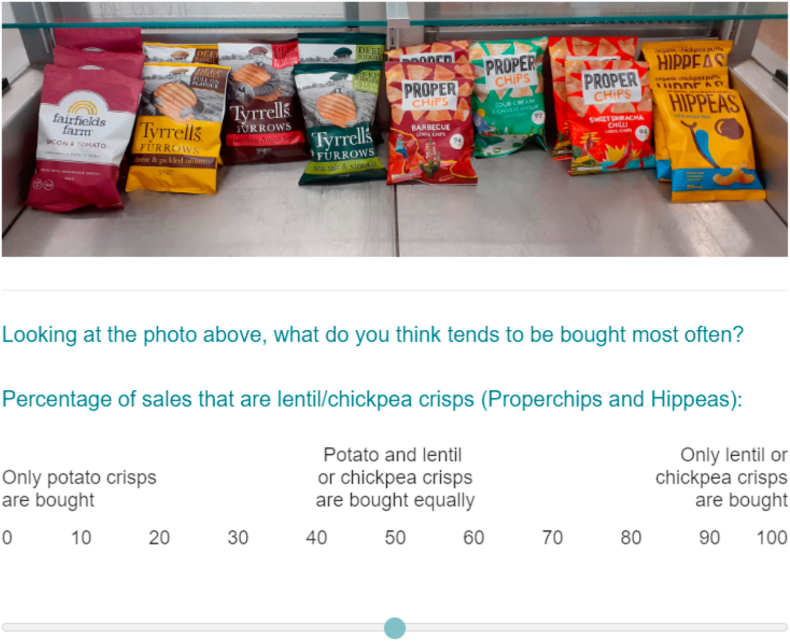
Example screenshot of perceived popularity question.

Participants were shown the Participant Information Sheet and asked to consent online. As part of an online questionnaire, each participant was presented with a series of images. Each image showed a shelving unit in a cafeteria displaying drinks or snacks. Participants were randomised to one of the availability conditions for each set of food products (four category-level and four product-level). For each image, participants were asked to rate (via a slider question; see Fig. 3) the proportion of sales from the lower energy category/product vs. the higher energy category/product (“Looking at the photo above, what do you think tends to be bought most often?”). The slider scale was labelled “Percentage of sales that are [lower energy product/category name]”, with labels at 0 (“Only [higher energy product/category name] is bought”), 50 (“[higher energy product/category name] and [lower energy product/category name] are bought equally”) and 100 (“Only [lower energy product/category name] is bought”). For each image, participants were also asked which option they would buy to eat or drink right now as a measure of product selection. For the product-level trials, participants were asked to rate the quality of the products [with options from, e.g. “Crisps are far better”, “Crisps are quite a bit better”, “Crisps are a little better”, “No difference in quality”, “Popcorn is a little better”, “Popcorn is quite a bit better”, “Popcorn is far better”]. After being shown the series of images, participants completed measures on age, gender, highest educational qualification, postcode, household income and hunger.

##### 3.1.2.5 Analyses

###### 3.1.2.5.1 Primary analysis

To answer Research Questions 1 and 2, a multilevel regression predicted the perceived percentage of lower energy (over higher energy) item sales from ranges with (i) ^1^/_4_ lower energy and ^3^/_4_ higher energy products, and (ii) ^3^/_4_ lower energy and ^1^/_4_ higher energy products, compared to ^1^/_2_ lower energy and ^1^/_2_ higher energy products, when shelves are (a) fuller and (b) emptier, with interactions between availability and shelf-fullness, and random effects by participant. Covariates included age, gender, education, hunger, manipulation-level (category-level vs. product-level), and food type.

###### 3.1.2.5.2 Secondary analyses

To answer Research Question 3, a multilevel logistic regression predicted the selection of lower energy (over higher energy) foods from the perceived purchases of lower energy vs. higher energy foods by others. Analyses were conducted with random effects by participant, and covariates included age, gender, education, hunger, and food type.

For Research Question 4, a multilevel logistic regression predicted the selection of lower energy foods from ranges with (i) ^1^/_4_ lower energy and ^3^/_4_ higher energy products, and (ii) ^3^/_4_ lower energy and ^1^/_4_ higher energy products, compared to ^1^/_2_ lower energy and ^1^/_2_ higher energy products, with interactions by manipulation-level (category-level vs. product-level). Analyses were conducted for full-shelf trials only (matched to previous online studies of availability to allow comparisons, given the aim of this research question was to test the effect of the manipulation-level on the impact of availability), with random effects by participant. Covariates were the same as for the Research Question 3 analysis.

For our main analyses, we used p < 0.05 (two-tailed) to infer that there is a statistically significant effect. For the remaining analyses (secondary analyses: Research Questions 3 and 4) we used a p-value <0.005 (two-tailed), using a Bonferroni adjustment to account for the different hypotheses to be tested (p = 0.05/9).

## 4 Study 1 Results

The mean age of the 2331 participants was 47.1 years (s.d. 16.9; range 18–92), and 51.3% were female (1191 females, 1132 males; 8 reported gender as ‘Other’). In terms of education, 49.9% (n = 1163) reported having qualifications that placed them in the higher education group, and 50.1% (n = 1168) in the lower education group.

### 4.1 Primary Research Questions

The results of the multilevel regression suggest that when changing from ^1^/_2_ lower energy availability to ^1^/_4_ lower energy in the fuller condition, the proportion of expected sales for lower energy items dropped by 3.1 percentage points (95% CIs: -4.2, −2.1; p < 0.001), and when changing from ^1^/_2_ lower energy availability to ^3^/_4_, the proportion of sales expected to be lower energy foods increased by 3.1 percentage points (95% CIs: 2.1, 4.2; p < 0.001). There was no evidence of a main effect of shelf-fullness (coefficient for emptier, compared to fuller: 0.03, 95%CIs: -1.0, 1.1; p = 0.951). There were significant interaction effects (coefficient for ^1^/_4_ lower energy & emptier shelf: 7.3, 95%CIs: 5.8, 8.8; p < 0.001; coefficient for ^3^/_4_ lower energy & emptier shelf: -8.1, 95%CIs: -9.6, −6.6; p < 0.001) – see Supplementary Table S1 for full model coefficients.

**Fig. 4:**
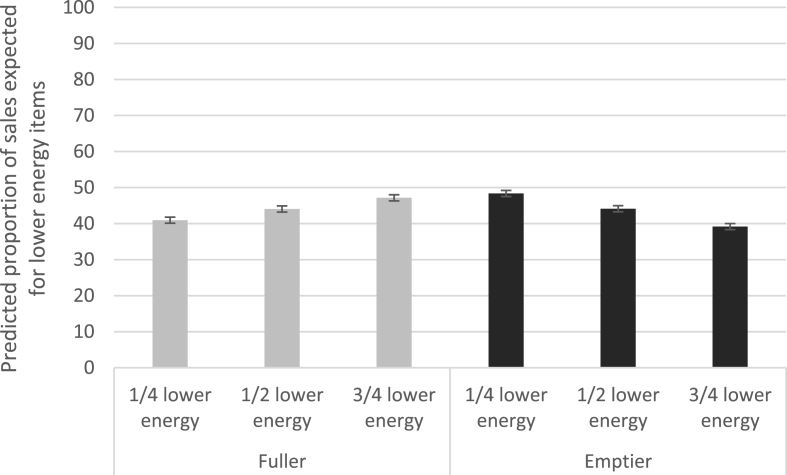
Proportion of expected sales for lower energy (over higher energy) options in each availability x shelf-fullness condition (Model predictions; Error bars show 95%CIs).

Fig. 4 shows the predicted proportion of sales expected for lower energy (over higher energy) options in each availability x shelf-fullness condition following the multilevel regression analyses. This shows that when the image showed a fuller shelf, the expected proportion of lower energy sales increased with an increasing proportion of lower energy items in the image. In contrast, when the image showed an emptier shelf, the expected proportion of lower energy sales decreased with an increasing proportion of lower energy items in the image. These differences were statistically significant but relatively small (between 41% at ^1^/_4_ lower energy to 47% at ^3^/_4_ lower energy for fuller shelves, and 48% at ^1^/_4_ lower energy to 39% at ^3^/_4_ lower energy for emptier shelves).

### 4.2 Secondary research question 3

**Fig. 5:**
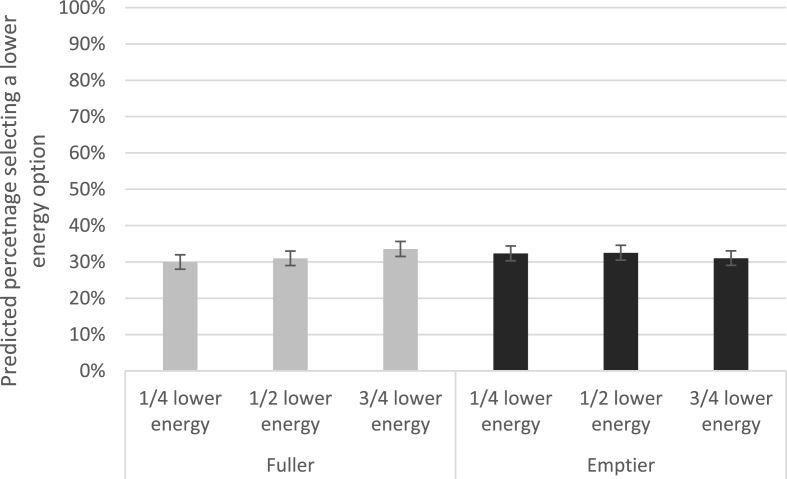
Percentage selecting a lower energy (over higher energy) option in each availability x shelf-fullness condition (Model predictions; Error bars show 95%CIs).

The results of the multilevel regression suggest that when changing lower energy availability in the fuller condition, there was no evidence of a difference in selection of lower energy products (^1^/_4_ lower energy: odds ratio 0.95; 95% CIs: 0.84, 1.07; p = 0. 40; ^3^/_4_ lower energy: odds ratio 1.14; 95% CIs: 1.01, 1.28; p = 0.04). There was no significant main effect of shelf-fullness (odds ratio for emptier, compared to fuller shelves: 1.07, 95%CIs: 0.94, 1.20), or interactions between lower energy availability and shelf-fullness (^1^/_4_ lower energy & emptier: odds ratio 1.04, 95%CIs: 0.88, 1.24; p = 0.64; ^3^/_4_ lower energy & emptier: odds ratio 0.82, 95%CIs: 0.69, 0.98; p = 0.03) (N.B. results were considered significant at p < 0.005 for secondary analyses; see Supplementary Table S2 for full model coefficients). Fig. 5 shows the predicted percentages, with very small differences predicted – from 30% to 34% with increasing lower energy availability for the fuller condition, and 32%–31% for the emptier condition.

### 4.3 Secondary research question 4

Modelling suggested no significant main effect of manipulation level (odds ratio for within-category, compared to between-category: 0.99; 95%CIs: 0.84, 1.18; p = 0.944) or interactions with availability (^1^/_4_ lower energy & within-category: odds ratio: 1.17; 95%CIs: 0.92, 1.50; p = 0.202; ^3^/_4_ lower energy & within-category: odds ratio: 1.24; 95%CIs: 0.97, 1.50 p = 0.089). See Supplementary Table S3 for full model results.

#### 4.3.1 Exploratory analyses

Exploratory analyses examined whether the perceived popularity of lower energy option(s) predicted selection of a lower energy option. A multilevel logistic regression suggested that for each additional percentage point increase in perceived popularity of lower energy options, the odds of selecting a lower energy option increased by 1.034 (95%CIs: 1.032, 1.036; p < 0.001; see Supplementary Table S4 for full model).

## 5 Study 1 Discussion

The results showed that with a fuller shelf, the expected proportion of lower energy sales increased with an increasing proportion of lower energy items. In contrast, with an emptier shelf, this pattern reversed so that the expected proportion of lower energy sales decreased with an increasing proportion of lower energy items. This suggests that the availability of lower energy products does impact on the perceived popularity (albeit predicting a relatively small change in expected sales). Moreover, this impact varies depending on whether the shelves were fuller or emptier. When shelves were fuller, a higher proportion of lower energy options led to greater perceived popularity, whereas when shelves were emptier (implying that others had previously selected some of these products), lower proportions of lower energy options led to greater perceived popularity.

The shelf-fuller and category-level conditions reflect those used in previous studies (Pechey & Marteau, 2018; Pechey et al., 2021), which suggested that as the proportion of lower energy options increase, the likelihood of selecting a lower energy option increases. In the current study, there was no evidence of a difference in selection of lower energy products when changing availability in either the fuller or emptier conditions. An exploratory analysis, however, suggested a small but significant increase in the odds of selecting a lower energy option for each additional percentage point increase in perceived popularity of lower energy options, supporting the potential role of perceived popularity as a mediator of the impact of availability on food selection.

One possible factor in the lack of any impact on lower energy option selection – in contrast to previous studies – is the inclusion of both product-level and category-level manipulations. Making changes at the product-level (rather than the category-level) does not alter the product range, so this condition minimises the effects of availability due to different preferences between products (another possible mediator of the effects of availability on food selection). Indeed, in order to minimise these differences, the product-level manipulation was used in Study 2. Importantly, Study 1 results suggested that there was no difference in the impact of availability on perceived popularity between making changes at the product-level rather than the category-level.

One potential limitation of Study 1 was that participants were explicitly asked about popularity before their selections, which could alter their behaviour. In addition, this study was conducted online and measured hypothetical selection, so participants did not receive their selected snack. Study 2 (below) aimed to address these limitations, examining real-life selections without explicitly drawing participants’ attention to item popularity.

## 6 Study 2: Laboratory study

### 6.1 Study 2 Aim

To examine the role of perceived popularity – as a marker of descriptive social norms – in determining the effects of relative availability interventions when presentation and selection involve physically-present options.

#### 6.1.1 Study 2 research question

To what extent does manipulating the implied popularity of lower energy vs. higher energy foods alter the impact of changing the relative availability of lower energy (over higher energy) foods?

##### 6.1.1.1 Participants

A sample of UK adults was recruited from a market research agency (Roots Research) between February and March 2020. The study aimed to recruit comparable numbers of males and females, and comparable numbers of participants with no A-levels or equivalent (categorised as lower education) and 2 or more A-levels or higher (higher education). Those who were studying for A-levels were excluded from participation.

This was an add-on to an existing study, with sample size (n = 279) having being determined for that study (Clarke et al., preprint). Power calculations were conducted using G*Power (3.1.9.2), to determine power to replicate the effect of availability found in a previous laboratory study (Pechey et al., 2021) – i.e. the effect of availability with a fuller display. As the fuller display represented half the conditions in the current study, the sample size was taken to be 139 (i.e. half the sample of 279). Calculations were based on a logistic regression with a 2-group comparison, with participants equally divided between groups, and an alpha of 0.025 to adjust for investigating two main effects. R-squared from other X (0.025) and the baseline proportion choosing a lower energy option (21%; from the predominantly higher energy condition) were taken from the previous study (Pechey et al., 2021). This sample size gave us a power of 0.81 to detect an effect equating to the change in the proportion of lower energy options selected to match that found in the equal numbers of lower energy and higher energy options condition in Pechey et al. (44%; odds ratio: 2.95) – and a power of 0.99 if this change was equivalent to the proportion of lower energy options chosen in the predominantly lower energy condition in Pechey et al. (66%; odds ratio: 7.30) (one-tailed).

These studies were terminated early on March 16, 2020, due to the COVID-19 pandemic. Analyses were therefore conducted on data from the smaller number of participants who had already completed the study procedures (n = 140). One participant of the 140 declined to take part in this add-on study, so the final sample size for this study was 139. The revised power calculations, based on half the achieved sample size (n = 69), give a power of 0.51 to detect an odds ratio of 2.95 and of 0.96 for an odds ratio of 7.30.

##### 6.1.1.2 Design

The study altered the range and presentation of snacks offered as “an end-of-study thank you”. It was a between-subjects laboratory study: 2 × 2 Availability (Predominantly higher energy options vs. Predominantly lower energy options) x Implied popularity (Greater consumption by others implied for option with more units remaining vs. Greater consumption by others implied for option with fewer units remaining).

For availability, the predominantly higher energy options condition comprised 6 units of the higher energy option vs. 3 units of the lower energy option, whereas the predominantly lower energy options condition comprised 3 units of the higher energy option vs. 6 units of the lower energy option.

For popularity, following on from the results of Study 1, it was hypothesised that greater consumption by others was implied for options that have more units when options are presented on a fuller tray (i.e. Fuller & ^2^/_3_ > Fuller & ^1^/_3_), whereas greater consumption by others was implied for options that have fewer units remaining when options are presented on an emptier tray (Emptier & ^1^/_3_ > Emptier & ^2^/_3_).

Options were set out so that nearest unit of each was equally close to participant, to avoid any impact due to a proximity effect (see Table 2). In order to limit any impact of differential preferences, the options offered were multiple units of the same product – i.e. just one type of lower energy and higher energy product was offered.

**Table 2:** Tray layouts in each condition.

		Relative Availability	
		Predominantly higher energy options	Predominantly lower energy options
**Implied popularity**	Tray Fuller: *Greater consumption by others implied for option with more units*	Group 1	Group 2
	Tray Emptier: *Greater consumption by others implied for option with fewer units*	Group 3	Group 4

N.B. A larger tray was used in the Tray Emptier conditions; The bars' orientation was also changed in this condition due to being unable to source matching trays in the sizes that would otherwise be required.

Randomisation was based on computer generated random sequences with Stata version 15 (by RP). Participants were allocated to conditions by the research team, in order of participation. Researchers were not blinded to allocation.

##### 6.1.1.3 Materials

Healthiness of food options was defined as in Study 1. Options were selected to differ in perceived healthiness, and to match in terms of familiarity, quality, perceived serving size and type of snack option (savoury or sweet) (Pechey & Marteau, 2018). Options were also matched as closely as possible in terms of actual size.

Lower energy options used were Alpen Light bar (Cherry Bakewell; 66 kcal; 19 g) and Pulsin Fruity Oat Bar (Orange Choc Chip; 97 kcal; 25 g). Higher energy options were Sainsbury's Taste the Difference Billionaire slice (323 kcal; 60 g) and Reese's Nutrageous bar (241 kcal; 47 g). Options were intended to be paired in a 2 × 2 manner, to minimise the influence of a particular option or pairing of options. Due to the early termination of this study, only two pairings were used: Alpen vs. Billionaire slice and Pulsin vs. Nutrageous. A sensitivity analysis was conducted to control for the options offered.

##### 6.1.1.4 Procedure

Participants were invited to take part in a prior, unrelated study (Clarke et al. (preprint); https://osf.io/dj3c6/; https://www.isrctn.com/ISRCTN66774780). As participants were ostensibly only recruited to this unrelated study, they consented to take part in a study examining how different plates and glasses can affect the visual appeal and attractiveness of food and drink. Measures completed in the prior study were considered unlikely to impact on subsequent behaviour in the current study: participants were asked to serve themselves (in a randomised order) their typical amount of (a) rice, using 3 plate sizes x 2 plate shapes and (b) wine, using 3 wine glass sizes x 2 wine bottle sizes. Given that this was a within-subjects design, all participants completed the same tasks prior to the current study. Participants did not consume any food or drink in this prior study.

After completion of the unrelated study, and prior to debriefing, participants were presented with a tray of food options and asked to select a snack, ostensibly as a thank you for their participation. The arrangement of snacks on the tray differed in line with participants’ allocated condition. The researcher left the room while participants chose a snack, to minimise potential social desirability effects. The researcher recorded which snack each participant selected, or if they declined to take a snack. Participants were then given an information sheet on the current study and asked to consent for their data to be used for the study purpose. If participants consumed their snack immediately their empty wrappers were removed from the testing room prior to the next participant arriving.

Demographic questions on age, gender and education completed at the start of the prior study were used in the analyses of the current study.

##### 6.1.1.5 Analyses

To test the primary research question, a logistic regression was conducted predicting whether participants selected a lower energy (over higher energy) snack option (participants who declined to take a snack were excluded from this analysis). Key predictors were availability condition, implied popularity condition and their interaction, and covariates were age, gender and education.

A secondary analysis was also planned to test whether there was a difference in declining to select a snack by study condition. This was a logistic regression predicting whether or not participants selected a snack from availability and implied popularity conditions, with covariates as above.

Given the early termination of this study, a sensitivity analysis was conducted to control for the food options offered to investigate whether this affected the results.

## 7 Study 2 Results

The mean age of the 139 participants was 40.6 (s.d. 14.1; range 18–71). They were predominantly female (68.3%; n = 95; the remainder identified as male), and most reported qualifications that would place them in the higher education group (80.4%; n = 111; data on education was missing for one participant).

Ten participants declined to choose a snack (two in the fuller tray & ^2^/_3_ lower energy availability condition and two in the fuller & ^1^/_3_ lower energy availability condition; three in the emptier tray & ^2^/_3_ lower energy availability condition and three in the emptier & ^1^/_3_ lower energy availability condition) (see supplementary materials: CONSORT flow diagram).

### 7.1 Primary analysis

In terms of selecting a lower energy snack option, 34.5% (n = 10) participants in the fuller tray & ^2^/_3_ lower energy availability condition made lower energy selections, compared to 15.4% (n = 6) in the fuller tray & ^1^/_3_ lower energy availability condition. In the emptier tray & ^2^/_3_ lower energy availability condition, 27.8% (n = 10) selected a lower energy option, compared to 36.0% (n = 9) in the emptier tray & ^1^/_3_ lower energy availability condition.

**Fig. 6:**
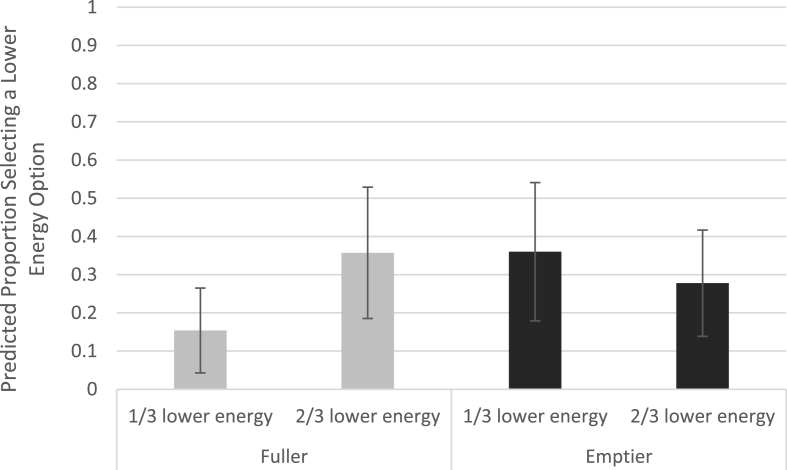
Predicted probability of selection of a lower energy snack option by availability condition and tray fullness (Error bars show 95% CIs).

Logistic regression analyses suggested that when the tray was fuller, participants were 3.3 times (95%CI: 0.99, 10.9; p = 0.053) more likely to select a lower energy snack when ^2^/_3_ options were lower energy than when ^2^/_3_ options were higher energy. When ^2^/_3_ options were higher energy, participants were 3.6 times (95%CI: 1.4, 12.4; p = 0.042) more likely to select a lower energy option when the tray was emptier compared to when the tray was fuller. The interaction between availability and tray fullness did not reach statistical significance (^2^/_3_ lower energy & Emptier tray: odds ratio: 0.20; 95%CI: 0.04, 1.1; p = 0.07). Fig. 6 shows the predicted pattern of lower energy option selections from the logistic regression model (see Supplementary Table S5 for full model results).

The secondary analysis predicting whether or not participants selected a snack from availability and implied popularity conditions was not conducted, due to small numbers declining.

#### 7.1.1 Sensitivity analysis

The sensitivity analysis including the foods offered showed very similar odds ratios (For ^2^/_3_ lower energy availability, vs. ^1^/_3_: odds ratio: 3.6, 95%CIs: 1.1, 12.0; p = 0.041; For Emptier tray vs. fuller: odds ratio: 3.5, 95%CIs: 0.999, 12.0; p = 0.050; For the interaction term ^2^/_3_ lower energy*Emptier tray: odds ratio: 0.19, 95%CIs: 0.03, 1.08; p = 0.062).

## 8 Study 2 Discussion

The results from Study 2 on lower energy snack selection showed a similar pattern to those for Study 1 for perceived popularity, namely that for fuller trays, a greater proportion of participants selected a lower energy option with increased lower energy availability, whereas for emptier trays, a lower proportion selected a lower energy option with increased lower energy availability. This suggests that the layout (implying differential popularity) of the same number of lower energy and higher energy products may affect the likelihood of a lower energy product being selected. However, the confidence intervals for the main effects and interactions in the logistic regression model were wide and crossed zero.

Due to the early termination of the study, the sample size was smaller than intended, and randomisation to groups was not evenly distributed, with 31 participants in the fuller tray & ^2^/_3_ lower energy availability condition; 41 in the fuller & ^1^/_3_ lower energy availability condition; 39 in the emptier tray & ^2^/_3_ lower energy availability condition; and 28 in the emptier & ^1^/_3_ lower energy availability condition. As such, while this provides an initial suggestion that the predicted interaction between availability and display layout may be found in laboratory settings, where participants make a real food selection, a larger study may reduce the very wide confidence intervals around the estimates. In addition, carrying out similar studies in settings without a researcher present would be beneficial, to reduce any potential social desirability effects, while running the study independently rather than as an add-on would rule out any impact of the prior studies on behaviour.

## 9 General Discussion

Together, these studies suggest that social norms – as implied by popularity – are one mechanism by which food selection is influenced by the proportion of lower energy food that is available. In particular, Study 1 shows the first step necessary for possible mediation, whereby altering the availability of lower energy food impacts on its perceived popularity. Study 2 in turn suggests that the popularity of lower energy foods (as implied by the relative availability of lower energy options with different tray fullness) could alter lower energy food selection – the second step of this potential mediatory pathway. The exploratory analysis conducted for Study 1 also suggested that perceived popularity of lower energy options predicted selection of lower energy options, supporting the idea of popularity as a potential mediator. These findings tie in with previous studies suggesting that environmental cues implying the behaviour of previous participants influence selections (Prinsen et al., 2013).

In addition, these studies highlight the potential moderation of the impact of product availability depending on product layout. The studies suggest the influence of layout may occur through implying perceived popularity – Study 1 indicating that how products are displayed alters the impact of lower energy food availability on perceived popularity, while Study 2 suggests that implying lower energy foods are more popular by altering the layout of products could increase lower energy food selection. If replicated in a larger study, this could have implications for how and in which contexts availability interventions would be most likely to have the desired impact on behaviour.

### 9.1 Strengths and limitations

This set of studies offers the first test of the role of social norms as a possible mechanism underlying the impact of altering the availability of lower energy vs. higher energy options. The conclusions from the studies are strengthened by a similar pattern of results by availability and layout in both online and laboratory settings. These studies recruited different sets of participants and used different food options, suggesting these effects may be reasonably robust.

While the studies are consistent with social norms acting as a potential partial mediator of the impact of altering availability on food selection, these findings are not able to fully assess the degree to which this occurs. Whilst it would be possible to conduct formal mediation analyses for Study 1, participants were already primed to product popularity – by being explicitly asked to estimate this – prior to their food selections, so such analyses would be likely to overestimate the impact of the role of social norms. In contrast, in Study 2 the popularity of items was never made explicit to, or estimated by, participants, but instead implied by the arrangement of the options. As such, neither study was designed to assess the whole of the potential mediatory pathway. Indeed, given that social norms may act as an unconscious bias in this mediatory pathway, it may prove difficult to assess this whole pathway in one study. A second key limitation, as noted above, was Study 2 being cut short, resulting in a smaller and unbalanced sample and a large degree of uncertainty around the estimates of effects. This limits the confidence with which conclusions can be drawn from this set of results, and highlights the need for replication of these effects in a larger and more diverse sample, which would allow more precise estimates to be obtained.

### 9.2 Implications for research and policy

When altering the availability of products, the product range is nearly always altered. As such, one mechanism that might underlie availability interventions is the relative preferences that exist between the available options. However, if an effect of availability is replicated in a study using a product-level manipulation (as in Study 2), this would show that altering product availability can be effective in the absence of altering the product range, i.e. without altering the preferences between the options offered. This is of particular interest, given that preferences for lower energy vs. higher energy options may vary by socioeconomic group (Pechey et al., 2015; Turrell, 1998). As such, it is possible that availability interventions where the effects are driven by underlying preferences may have the potential to exacerbate health inequalities that result from diet. The findings from this set of studies are the first to suggest that preferences are not the only mechanism that underlies availability interventions, but that social norms also play a role. Establishing the mechanisms that might underlie availability interventions is key to enabling these promising interventions to be optimally implemented to improve the healthiness of diets across all groups.

These studies offer a first test of the role of social norms in availability interventions, but also suggest that setting may act as a moderator, as the popularity of different options may be implied in a different way or to a different extent within a given context. Study 1 looked at perceived popularity in a context akin to purchasing in a cafeteria. Further research could explore this effect in different purchasing contexts – such as vending machines and supermarket settings, where expectations of machine or shelf layout and restocking are likely to vary. In addition, investigating whether existing social norms may be stronger – and perhaps less easily manipulated – for higher energy rather than lower energy options, given higher energy options are often more familiar, could have implications for how best to operationalise availability interventions. Finally, the role of social norms as a potential mediator of availability on food selection could be compared for food options with varying discrepancies in preference – i.e. little discrepancy between options versus wide discrepancy – to establish the extent to which social norms might be able to counteract the impact of preferences. This may tie in with findings from studies looking at social norms, whereby social norms have been found not to influence selection of less palatable but healthy cookies over less-healthy cookies (Pliner & Mann, 2004).

## 10 Conclusion

These studies provide novel evidence that social norms may act as a partial mediator of the impact of availability on food selection. In addition, they suggest that the effect of availability may vary in different contexts, moderated by display layout through its impact on perceived product popularity. This could have implications for how and in which contexts availability interventions would be most likely to have the desired impact on behaviour.

## Declaration of competing interest

None.

## Authors’ contributions

RP, NC, GJH and TMM designed the study. NC, EP, MV and RP conducted the study. RP analysed the data and drafted the manuscript. NC, EP, MV, GJH and TMM provided critical revisions to the manuscript. All authors read and approved the final manuscript.

## Funding sources

RP is supported by a 10.13039/100010269Wellcome Trust Research Fellowship in Society and Ethics [106679/Z/14/Z]. TMM and GJH hold a Collaborative Award in Science from 10.13039/100010269Wellcome Trust (Behaviour Change by Design: 206853/Z/17/Z to Theresa Marteau, Paul Fletcher, Gareth Hollands and Marcus Munafò). The funders had no role in the study design, data collection, analysis, or interpretation. For the purpose of Open Access, the author has applied a CC BY public copyright licence to any Author Accepted Manuscript version arising from this submission.

## Ethics approval

Ethical approval was obtained from the Cambridge Psychology Research Ethics Committee (PRE.2019.100). Written consent was obtained from all participants.

## Availability of data

The datasets generated during the current study are available in the Open Science Framework: https://osf.io/qkh8c/files/.
