# Supplementary material for: Impact of altering the available food options on selection: Potential mediation by social norms
Source: Appetite. 2021 Sep 1;164:105245. doi: 10.1016/j.appet.2021.105245 (PMC8214103; doi:10.1016/j.appet.2021.105245)
Supplement: Multimedia component 1 [file mmc1.docx]

**SUPPLEMENTARY MATERIALS**

**Impact of altering the available food options on selection: potential mediation by social norms**

**Table S1**: Study 1: Results from multilevel regression: Proportion of sales expected for lower energy items

|  | | Coefficient | 95% Confidence Intervals | | p-value |
| --- | --- | --- | --- | --- | --- |
|  |  |  | Lower | Upper |  |
| Availability condition  (Ref: ^1^/_2_ lower energy) | ^1^/_4_ lower energy | -3.11 | -4.18 | -2.05 | <0.001 |
|  | ^3^/_4_ lower energy | 3.14 | 2.08 | 4.21 | <0.001 |
| Fullness condition (Ref: Fuller) | Emptier | 0.03 | -1.03 | 1.09 | 0.951 |
| Availability x Fullness | ^1^/_4_ lower energy & Emptier | 7.33 | 5.83 | 8.83 | <0.001 |
|  | ^3^/_4_ lower energy & Emptier | -8.07 | -9.57 | -6.56 | <0.001 |
| Age | | -0.08 | -0.12 | -0.05 | <0.001 |
| Gender (Ref: Female) | Male | 0.63 | -0.41 | 1.66 | 0.235 |
|  | Other | 0.99 | -7.59 | 9.57 | 0.820 |
| Education (Ref: Higher) | Lower | 0.50 | -0.50 | 1.51 | 0.326 |
| Hunger | | -1.33 | -1.71 | -0.94 | <0.001 |
| Food type  (Ref: Savoury snacks) | Drinks | 10.57 | 9.85 | 11.29 | <0.001 |
|  | Sweet snacks | 3.52 | 2.80 | 4.24 | <0.001 |
| Manipulation level  (Ref: Between-categories) | Within categories | 3.54 | 2.96 | 4.13 | <0.001 |
| Constant | | 42.44 | 40.67 | 44.21 | <0.001 |
|  | | | | | |
| Random-effects Parameters (id: Identity) | | | | | |
| var(_cons) | | 99.96 | 91.53 | 109.17 | - |
| var(Residual) | | 417.66 | 408.70 | 426.83 | - |

*18,648 observations from 2331 participants*

**Table S2.** Study 1: Multilevel logistic regression (Secondary Research Question 3): Selection of a lower energy item when asked which they would buy to eat right now

|  | | Odds ratio | 95% Confidence Intervals | | p-value |
| --- | --- | --- | --- | --- | --- |
|  |  |  | Lower | Upper |  |
| Availability condition  (Ref: ^1^/_2_ lower energy) | ^1^/_4_ lower energy | 0.95 | 0.84 | 1.07 | 0.403 |
|  | ^3^/_4_ lower energy | 1.14 | 1.01 | 1.28 | 0.039 |
| Shelf-fullness condition (Ref: Fuller) | Emptier | 1.07 | 0.94 | 1.20 | 0.307 |
| Availability x Shelf-fullness | ^1^/_4_ lower energy & Emptier | 1.04 | 0.88 | 1.24 | 0.635 |
|  | ^3^/_4_ lower energy & Emptier | 0.82 | 0.69 | 0.98 | 0.027 |
| Age | | 1.002 | 0.999 | 1.005 | 0.267 |
| Gender (Ref: Female) | Male | 0.72 | 0.65 | 0.80 | <0.001 |
|  | Other | 1.14 | 0.49 | 2.67 | 0.763 |
| Education (Ref: Higher) | Lower | 0.77 | 0.70 | 0.85 | <0.001 |
| Hunger | | 0.91 | 0.88 | 0.95 | <0.001 |
| Food type  (Ref: Savoury snacks) | Drinks | 4.90 | 4.51 | 5.33 | <0.001 |
|  | Sweet snacks | 1.47 | 1.35 | 1.59 | <0.001 |
| Manipulation level  (Ref: Between-categories) | Within categories | 1.14 | 1.07 | 1.22 | <0.001 |
| Constant | | 0.32 | 0.27 | 0.39 | <0.001 |
|  | | | | | |
| Random-effects Parameters (id: Identity) | | | | | |
| sd(_cons) | | 0.90 | 0.84 | 0.96 | - |

*18,648 observations from 2331 participants*

**Table S3.** Study 1: Multilevel logistic regression (Secondary Research Question 4): Selection of a lower energy item when asked which they would buy to eat right now

|  | | Odds ratio | 95% Confidence Intervals | | p-value |
| --- | --- | --- | --- | --- | --- |
|  |  |  | Lower | Upper |  |
| Availability condition  (Ref: ^1^/_2_ lower energy) | ^1^/_4_ lower energy | 0.88 | 0.74 | 1.05 | 0.168 |
|  | ^3^/_4_ lower energy | 1.02 | 0.85 | 1.21 | 0.862 |
| Manipulation-level (Ref: Between-category) | Within-category | 0.99 | 0.84 | 1.18 | 0.944 |
| Availability x Manipulation-level | ^1^/_4_ lower energy & Within-category | 1.17 | 0.92 | 1.50 | 0.202 |
|  | ^3^/_4_ lower energy & Within-category | 1.24 | 0.97 | 1.50 | 0.089 |
| Age | | 1.002 | 0.998 | 1.006 | 0.278 |
| Gender (Ref: Female) | Male | 0.73 | 0.65 | 0.83 | <0.001 |
|  | Other | 0.64 | 0.22 | 1.92 | 0.429 |
| Education (Ref: Higher) | Lower | 0.81 | 0.72 | 0.92 | 0.001 |
| Hunger | | 0.93 | 0.89 | 0.97 | 0.002 |
| Food type  (Ref: Savoury snacks) | Drinks | 5.05 | 4.47 | 5.72 | <0.001 |
|  | Sweet snacks | 1.56 | 1.38 | 1.76 | <0.001 |
| Constant | | 0.32 | 0.25 | 0.40 | <0.001 |
|  | | | | | |
| Random-effects Parameters (id: Identity) | | | | | |
| sd(_cons) | | 0.90 | 0.81 | 0.99 | - |

*9,306 observations from 2327 participants [Emptier condition trials were excluded from this analysis; 4 participants were not randomised to any Fuller condition images]*

**Table S4.** Study 1: Multilevel logistic regression (Exploratory analysis)*:* Selection of a lower energy item when asked which they would buy to eat right now

|  | | Odds ratio | 95% Confidence Intervals | | p-value |
| --- | --- | --- | --- | --- | --- |
|  |  |  | Lower | Upper |  |
| Perceived popularity of lower energy option(s) | | 1.034 | 1.032 | 1.036 | <0.001 |
| Age | | 1.004 | 1.001 | 1.008 | 0.006 |
| Gender (Ref: Female) | Male | 0.67 | 0.60 | 0.74 | <0.001 |
|  | Other | 1.02 | 0.41 | 2.55 | 0.958 |
| Education (Ref: Higher) | Lower | 0.74 | 0.67 | 0.82 | <0.001 |
| Hunger | | 0.95 | 0.91 | 0.99 | 0.008 |
| Food type  (Ref: Savoury snacks) | Drinks | 3.99 | 3.66 | 4.35 | <0.001 |
|  | Sweet snacks | 1.35 | 1.23 | 1.48 | <0.001 |
| Manipulation level  (Ref: Between-categories) | Within categories | 1.03 | 0.96 | 1.11 | 0.363 |
| Constant | | 0.075 | 0.062 | 0.091 | <0.001 |
|  | | | | | |
| Random-effects Parameters (id: Identity) | | | | | |
| sd(_cons) | | 0.932 | 0.873 | 0.994 | - |

*18,264 observations from 2331 participants*

**Table S5.** Study 2: Logistic regression predicting selection of a lower energy snack option

|  | Odds Ratio | 95% Confidence  Intervals | | p-value |
| --- | --- | --- | --- | --- |
| Availability (^2^/_3_ lower energy) | 3.27 | 0.99 | 10.88 | 0.053 |
| **Layout (emptier)** | **3.61** | **1.05** | **12.42** | **0.042** |
| Availability x Layout  (^2^/_3_ lower energy & emptier) | 0.20 | 0.04 | 1.11 | 0.066 |
| Age | 1.01 | 0.98 | 1.05 | 0.394 |
| Gender (female) | 3.37 | 1.17 | 9.68 | 0.024 |
| Education (lower) | 1.93 | 0.67 | 5.50 | 0.221 |
| Constant | 0.03 | 0.01 | 0.19 | 0.000 |

*128 observations; Pseudo R-squared: 0.0929*

**Table S6**. Study 2; Sensitivity analysis: Logistic regression predicting selection of a lower energy option, controlling for the food options offered

|  | Odds Ratio | 95% Confidence  Intervals | | p-value |
| --- | --- | --- | --- | --- |
| **Availability (^2^/_3_** **lower energy)** | **3.56** | **1.05** | **12.03** | **0.041** |
| Layout (emptier) | 3.47 | 0.999 | 12.03 | 0.050 |
| Availability x Layout  (^2^/_3_ lower energy & emptier) | 0.19 | 0.03 | 1.08 | 0.062 |
| Age | 1.01 | 0.98 | 1.04 | 0.428 |
| Gender (female) | 3.34 | 1.16 | 9.61 | 0.025 |
| Education (lower) | 1.96 | 0.68 | 5.64 | 0.214 |
| Foods offered  (Pulsin & Nutrageous) | 1.63 | 0.70 | 3.81 | 0.259 |
| Constant | 0.03 | 0.00 | 0.16 | 0.000 |

*128 observations; Pseudo R-squared: 0.1015; BIC provides support for using simpler model – i.e. not including the variable accounting for the food options offered (difference of 3.561)*
